# An immunometabolism‐related signature for renal clear cell carcinoma diagnosis and therapeutic target

**DOI:** 10.1002/ccs3.70047

**Published:** 2025-09-17

**Authors:** Guofan Hu, Jian Liang, Meiling Feng, Hansheng Lin, Jingwei He

**Affiliations:** ^1^ Department of Urology Guangdong Medical University Zhanjiang China; ^2^ Department of Urology Yangjiang People's Hospital Yangjiang China

**Keywords:** biomarkers, immunometabolism, kidney renal clear cell carcinoma, machine learning, single‐cell analysis

## Abstract

Kidney renal clear cell carcinoma (KIRC) lacks sensitive early diagnostic markers and effective therapeutic guidance. Given the tight crosstalk between tumor metabolism and immunity, we investigated immunometabolism for biomarker discovery. Transcriptomes from The Cancer Genome Atlas (TCGA) and Gene Expression Omnibus were integrated. Immunometabolism‐related genes were screened by weighted gene co‐expression network analysis and differential expression, followed by three machine learning algorithms (least absolute shrinkage and selection operator, Support Vector Machine–Recursive Feature Elimination (SVM‐RFE), and random forest) to select features and build a diagnostic model. Performance was validated in external cohorts. Multi‐omics correlation, immune infiltration, drug‐sensitivity, and survival analyses were conducted. Functional assays were performed in vitro and in vivo. Six biomarkers—ABCB1, Acyl‐CoA Dehydrogenase Short/Branched Chain (ACADSB), PLA2G6, AKR1C3, PANK1, and Lactate Dehydrogenase B (LDHB)—were identified. The model showed strong discrimination (AUC 0.976 in TCGA; 0.902 in GSE126964; and 0.916 in GSE36895). The genes correlated with immune checkpoints, cytokine signaling, T‐cell infiltration, and clinical parameters. Drug analyses suggested cisplatin and sunitinib downregulated oncogenic targets. Silencing ABCB1 or AKR1C3, or overexpressing LDHB, suppressed KIRC cell proliferation and migration in vitro; LDHB overexpression combined with sorafenib significantly reduced tumor growth in vivo. We propose a robust immunometabolism‐based diagnostic model and six experimentally supported biomarkers for KIRC, providing mechanistic insight into tumor–immune interactions and potential avenues for personalized therapy.

## Graphical abstract

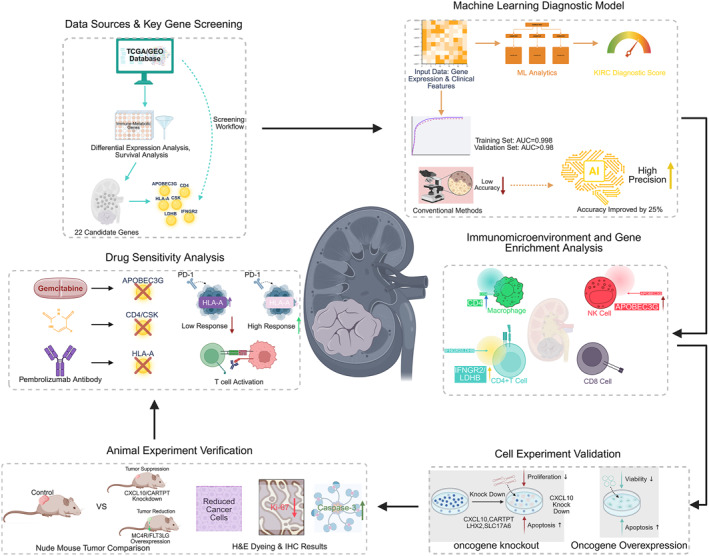

## 1 INTRODUCTION

Kidney renal clear cell carcinoma (KIRC), the most prevalent histological subtype of renal cell carcinoma (RCC), accounts for approximately 75% of all RCC cases. This malignancy exhibits substantial molecular and clinical heterogeneity, is typically associated with an unfavorable prognosis, and displays a strong tendency for distant metastasis.^1^, ^2^ Furthermore, patients with KIRC often experience poor survival outcomes.^3^, ^4^ Although targeted therapies and immunotherapies have become standard treatments for advanced KIRC and have significantly improved patient outcomes,^5^ drug resistance, and treatment‐related adverse effects remain major clinical challenges.^6^ Therefore, identifying novel diagnostic markers and therapeutic targets to enable early diagnosis and precision treatment of KIRC is of significant clinical importance.

Aberrant metabolism is a hallmark of cancer, and KIRC is often characterized as a “metabolic disease” due to its distinctive metabolic reprogramming.^7^ Glycolysis, fatty acid metabolism, and the tricarboxylic acid (TCA) cycle are notably altered, commonly driven by mutations in genes such as von Hippel‐Lindau (VHL), folliculin (FLCN), and transcription factor binding to Immunoglobulin Heavy Constant Mu (IGHM) enhancer 3 (TFE3).^7^, ^8^, ^9^ VHL loss activates hypoxia‐inducible factor‐1 (HIF‐1), enhancing the Warburg effect,^10^, ^11^, ^12^ whereas transforming growth factor‐beta and histone deacetylase 7 repress TCA cycle enzymes.^13^ These alterations promote tumor growth, affect prognosis, and impact therapeutic sensitivity.^8^ Concurrently, KIRC exhibits strong immunogenicity, with a tumor microenvironment (TME) rich in diverse immune cell populations.^14^ Immune checkpoint inhibitors such as nivolumab have shown efficacy, but variable responses and immune‐related adverse events limit their application.^6^, ^15^

Recent studies demonstrate that metabolic reprogramming and immune regulation are closely linked in KIRC. Tumor and immune cells compete for nutrients such as glucose and glutamine in the TME, impairing effector T cell activity while enabling regulatory T cells (Tregs) to sustain immunosuppressive functions through lipid oxidation.^16^ Moreover, immune cell fate and function depend on specific metabolic programs, highlighting metabolic checkpoints as potential therapeutic targets.^17^ Tumor‐derived metabolic stress, including reactive oxygen species and lipid peroxidation, can induce immunogenic cell death and enhance antitumor immunity.^18^ Integrating metabolic interventions with immunotherapy may overcome resistance and improve outcomes in KIRC patients.^19^ Understanding immunometabolism offers key insights into disease progression and supports the development of more effective therapeutic strategies.

Although existing studies have elucidated the critical roles of metabolic abnormalities and the immune microenvironment in KIRC,^20^, ^21^, ^22^, ^23^ research on diagnostic biomarkers for KIRC remains significantly limited. Current biomarkers lack sufficient sensitivity and specificity, failing to meet clinical demands, particularly for early diagnosis and personalized treatment.^24^, ^25^ Moreover, most studies are confined to single‐omics data or a single analytical approach, lacking systematic integration and multi‐dimensional validation of immunometabolism‐related genes.^26^, ^27^ These limitations hinder the accuracy of early KIRC diagnosis and its clinical applicability. Therefore, there is an urgent need to integrate multi‐omics datasets in combination with advanced analytical approaches to uncover diagnostic biomarkers associated with immunometabolic pathways, thereby addressing the current research gaps.

To address this, the study sought to comprehensively identify diagnostic biomarkers linked to immunometabolism in KIRC by leveraging multi‐omics datasets from The Cancer Genome Atlas (TCGA) and Gene Expression Omnibus (GEO), and employing weighted gene co‐expression network analysis (WGCNA) integrated with a range of machine learning techniques. A high‐performance predictive model was constructed and further validated through multiple levels of experimentation, including single‐cell transcriptomics, drug sensitivity databases, quantitative real‐time polymerase chain reaction (qRT‐PCR), western blot, and animal models. This research not only clarified the diagnostic value of the biomarkers from a data‐driven perspective but also validated their functional roles in KIRC development, providing both theoretical and experimental support for future clinical applications.

## 2 MATERIALS AND METHODS

### 2.1 Study design

As illustrated in Figure S1, this study was initiated by collecting transcriptomic and clinical datasets for KIRC from TCGA and GEO. Genes associated with immunometabolism were subsequently filtered and examined through WGCNA. Subsequently, differential expression analysis was combined with 12 machine learning algorithms to extract representative feature genes and develop a diagnostic prediction model. Finally, the model was validated across multiple independent validation datasets, followed by immune infiltration, drug sensitivity, and single‐cell analyses.

### 2.2 Data sources

Transcriptomic data related to KIRC were retrieved from the GEO database (https://www.ncbi.nlm.nih.gov/geo/), including GSE66272, GSE40435, GSE171306, GSE36895, and GSE53737. Transcriptomic and clinical data for KIRC were also acquired from the TCGA database (https://www.cancer.gov/ccg/research/genome‐sequencing/tcga). Furthermore, a total of 3431 genes associated with immunometabolic processes were collected from the GeneCards database (https://www.genecards.org/) (Table S1).

### 2.3 WGCNA and identification of differentially expressed genes

The WGCNA package in R (version 4.4.1) was applied to analyze the GSE66272 dataset, with immunometabolism‐related genes as the primary components for screening genes and identifying potential correlations with KIRC. Gene expression profiles within each co‐expression module were summarized, and both module significance and average gene significance were calculated. Modules with higher significance values were considered more important, and the most significant module was selected for further analysis. Differential expression analysis of immunometabolism‐related genes in the The Cancer Genome Atlas–Kidney Renal Clear Cell Carcinoma (TCGA‐KIRC) dataset was performed using the “Limma” package, with filtering criteria defined as |log_2_FC| > 1 and *p*‐value <0.05. The results were visualized, and a Venn diagram was used to analyze the intersection between WGCNA and differentially expressed genes (DEGs).

### 2.4 Immune infiltration analysis

To evaluate the infiltration levels of immune cells within the training cohort, single‐sample gene set enrichment analysis (ssGSEA) was employed. A total of 23 immune cell subtypes were quantified, including various activated B and T cells (CD4^+^ and CD8^+^), dendritic cells, natural killer (NK) and natural killer T (NKT) cells, myeloid‐derived suppressor cells, macrophages, monocytes, mast cells, eosinophils, neutrophils, γδ T cells, and several T cell subtypes such as Tregs, Th1, Th2, Th17, and follicular helper T cells.

### 2.5 Construction and validation of the diagnostic model

Twelve distinct machine learning algorithms were applied to analyze the intersecting genes, including least absolute shrinkage and selection operator, ridge regression, stepwise generalized linear model (Stepglm), extreme gradient boosting (XGBoost), random forest (RF), elastic net (Enet), partial least squares regression for generalized linear models (PlsRglm), gradient boosting machine, Naive Bayes, linear discriminant analysis, generalized linear model boosting (GlmBoost), and support vector machine. These algorithms were systematically implemented on the training cohorts (GSE53757 and GSE66272) to identify optimal features and construct predictive models through a 10‐fold cross‐validation strategy. Model performance was validated using independent datasets (GSE36895 and GSE40435). Predictive efficacy of the resulting nomogram was assessed using calibration curves, whereas clinical applicability was evaluated by decision curve analysis (DCA). The diagnostic value was determined using receiver operating characteristic (ROC) curves, and the accuracy of the diagnostic model was evaluated using a confusion matrix. Finally, the model was compared with two previously published KIRC diagnostic models, with *p*‐values <0.05 regarded as statistically significant.

### 2.6 Enrichment analysis of the diagnostic model

Functional enrichment analyses related to the diagnostic model were performed through the clusterProfiler package, emphasizing Gene Ontology (GO) annotations and Kyoto Encyclopedia of Genes and Genomes (KEGG) pathway analysis. DEGs were subjected to pathway screening, and key enrichment results were presented through bar plot visualizations. A significance threshold of *p* < 0.05 was applied for all analyses.

### 2.7 Single‐cell analysis

Single‐cell Ribonucleic Acid (RNA) sequencing analysis was carried out utilizing the Seurat package to explore genes involved in the diagnostic model and their interactions with immune cell populations.

### 2.8 Drug sensitivity analysis

Drug sensitivity profiling of genes involved in the diagnostic model was conducted using the OncoPredict package to identify potential therapeutic compounds targeting their molecular mechanisms. Additionally, the Tumor Immunotherapy Gene Expression Resource database (http://tiger.canceromics.org/#/immuneResponse) was utilized to investigate their sensitivity to PD‐L1.

### 2.9 Differential expression analysis of diagnostic model‐related genes

Differential expression analysis of diagnostic model‐associated genes was performed using the Limma package to assess their expression patterns in KIRC. Validation was conducted using immunohistochemical data from The Human Protein Atlas (HPA) database (https://www.proteinatlas.org/), based on representative sections from three normal kidneys and three KIRC tissues. These data were used for qualitative assessment of protein expression and not for quantitative analysis.

### 2.10 Cell lines and culture conditions

This study utilized the KIRC cell lines 786‐O and Caki‐1 and the normal renal tubular epithelial cell line HK‐2. The 786‐O and Caki‐1 cell lines were sourced from the Culture Collection of the Chinese Academy of Sciences (), and the HK‐2 cell line was obtained from the American Type Culture Collection (). Cells were cultured in RPMI‐1640 medium supplemented with 10% fetal bovine serum (FBS), 100 U/mL penicillin, and 100 μg/mL streptomycin at 37°C in a 5% CO_2_ humidified incubator. The culture medium was replaced every 2–3 days, and cells were passaged when confluency reached 70%–80%.

### 2.11 RNA extraction and qRT‐PCR

RNA was extracted utilizing TRIzol (Thermo Fisher Scientific), and quantified with a NanoDrop spectrophotometer (Thermo Fisher Scientific). First‐strand cDNA was synthesized using the EntiLink™ 1st Strand cDNA Synthesis SuperMix (GeneCopoeia). QRT‐PCR was conducted using the EnTurbo™ SYBR Green Polymerase Chain Reaction (PCR) SuperMix (GeneCopoeia) on a QuantStudio 6 Flex PCR system (Thermo Fisher Scientific). Glyceraldehyde‐3‐Phosphate Dehydrogenase (GAPDH) was used as the internal reference gene, and relative gene expression levels were calculated utilizing the 2^−ΔΔCT^ method. Primer sequences are listed in Table S2.

### 2.12 Western blot

Total proteins were extracted using Radio‐Immunoprecipitation Assay (RIPA) buffer with protease and phosphatase inhibitors (Beyotime), and quantified by Bicinchoninic Acid (BCA) protein assay (Thermo Fisher Scientific). Equal amounts of protein (30 μg) were resolved on 10% Sodium Dodecyl Sulfate–Polyacrylamide Gel Electrophoresis (SDS‐PAGE) gels and transferred to Polyvinylidene Fluoride (PVDF) membranes (Millipore). The membrane was blocked with 5% non‐fat milk in Tris‐Buffered Saline with Tween 20 (TBST) for 1 h, and the membranes were incubated overnight at 4°C with the following primary antibodies: Lactate Dehydrogenase B (LDHB) (1:5000), IFNGR2 (1:1000), CD4 (1:1000), Cytoskeleton‐Associated Protein (CSK) (1:20,000), HLA‐A (1:20,000), APOBEC3G (1:1000, all from Abcam), and the internal control GAPDH (1:2000, Proteintech) at 4°C overnight. The next day, the membrane was incubated with an HRP‐conjugated secondary antibody (1:5000, Abcam, ab6759) for 1 h, and protein bands were visualized using an Enhanced Chemiluminescence (ECL) substrate (Thermo Fisher Scientific). Band intensity was quantified using ImageJ software. Detailed antibody information is provided in Table S3.

### 2.13 Gene silencing and overexpression

shRNA sequences targeting IFNGR2, CD4, CSK, HLA‐A, and APOBEC3G were designed and synthesized by GenePharma, and corresponding shRNA expression vectors were constructed. A total of 50 nM shRNA was transfected into KIRC cell lines 786‐O and Caki‐1 using Lipofectamine™ 3000 Transfection Reagent (Invitrogen). After 48 h, total RNA and protein were isolated for qRT‐PCR and western blot analyses to evaluate gene knockdown efficiency. The shRNA sequences are listed in Table S4.

For LDHB functional studies, a pcDNA3.1(+)‐LDHB overexpression vector was generated by cloning LDHB cDNA into the pcDNA3.1(+) backbone using EcoRI and XhoI restriction sites. Cloning and sequence confirmation were performed by GenePharma. Transfection conditions mirrored those of the shRNA experiments. Forty‐eight hours post‐transfection, mRNA and protein expression levels were measured to assess overexpression efficacy. The plasmid sequence information is provided in the Supplementary Material.

### 2.14 Cell proliferation assay

Cell proliferation was assessed using the Cell Counting Kit‐8 (CCK‐8; Dojindo). Transfected cells were plated into 96‐well plates at a density of 2000 cells per well. After a 24‐h incubation, 10 μL of CCK‐8 reagent was added and incubated for 2 h. Absorbance was measured at 450 nm using a microplate reader (Bio‐Rad). Experiments were performed in triplicate.

### 2.15 Migration and invasion assays

Cell migration and invasion abilities were assessed using 8 μm pore‐size Transwell chambers (Corning). For the migration assay, 1 × 10^5^ transfected cells in a serum‐free medium were added to the upper chamber. In the invasion assay, the upper membranes were pre‐coated with Matrigel (BD Biosciences). Complete medium enriched with 10% FBS was placed in the lower chambers as a chemoattractant. Following a 24‐h incubation, non‐migrated or non‐invaded cells on the upper surface were carefully removed. The cells that traversed to the underside of the membrane were fixed with 4% paraformaldehyde, stained with crystal violet, and quantified under a light microscope.

### 2.16 Apoptosis detection

Cell apoptosis was detected using the Annexin V‐FITC/propidium iodide (PI) Apoptosis Detection Kit (Beyotime). After transfection, cells were harvested, washed twice, and resuspended in 500 μL of binding buffer. Subsequently, 5 μL of Annexin V‐FITC and 5 μL of PI were added, followed by incubation at room temperature in the dark for 15 min. Stained cells were subsequently analyzed on a flow cytometer (BD Biosciences), and data were processed using FlowJo software.

### 2.17 Nude mouse xenograft tumor model

To establish a KIRC xenograft model, 1 × 10^7^ transfected or non‐transfected 786‐O cells were subcutaneously injected into 4 to 6‐week‐old male Bagg Albino (BALB/c) nude mice. The experimental groups included a control group and a shRNA knockdown group, with six mice in each group. Tumor growth was monitored every 3 days, and volumes were estimated using the formula: volume = (length × width^2^)/2. After 4 weeks, mice were euthanized, and tumors were harvested and weighed. Histological evaluation was conducted via hematoxylin and eosin (H&E) staining, and Ki‐67 immunostaining was used to assess cellular proliferation. All procedures were approved by the Animal Ethics Committee of Yangjiang People's Hospital and conducted in compliance with ethical guidelines.

### 2.18 Drug sensitivity assay

In the in vivo experiments, nude mouse models were randomly assigned into three groups: shNC (negative control), shRNA + drug, and drug‐only groups. The drug (sorafenib, 20 mg/kg/day) was administered via oral gavage for 2 weeks, and tumor growth inhibition was monitored throughout the treatment period.

### 2.19 Statistical analysis

Data were analyzed using SPSS 24.0 (IBM) and presented as mean ± standard deviation. Student's *t*‐test was used for two‐group comparisons, and one‐way Analysis of Variance (ANOVA) followed by Tukey's post hoc test was applied for multiple group comparisons. Graphical presentations were generated in GraphPad Prism 8, and statistical significance was defined as *p* < 0.05.

## 3 RESULTS

### 3.1 Immunometabolism gene analysis and identification of immune infiltration characteristics

**FIGURE 1:**
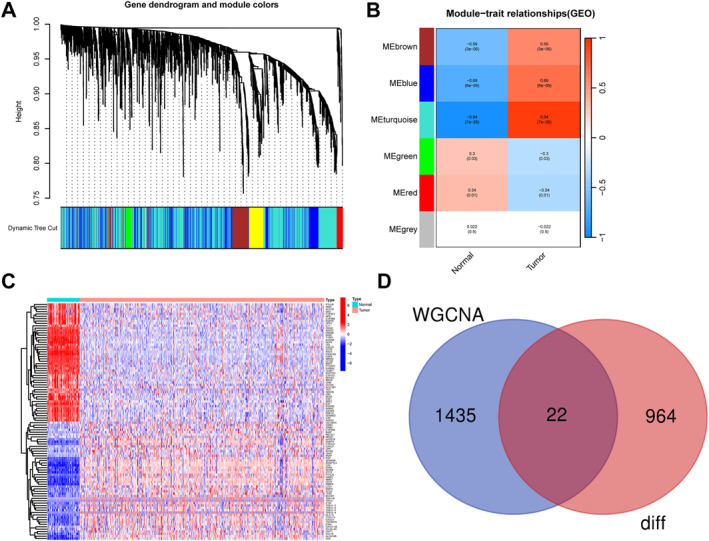
WGCNA and identification of DEGs. (A) Hierarchical clustering dendrogram based on WGCNA analysis. (B) ME heatmap showing the strong correlation between the ME turquoise module and immunometabolism features. (C) Heatmap of immunometabolism‐related genes in TCGA‐KIRC. (D) Venn diagram illustrating the intersection of genes from the ME turquoise module and DEGs. DEG, differentially expressed gene; ME, Module Eigengene; TCGA‐KIRC, The Cancer Genome Atlas–Kidney Renal Clear Cell Carcinoma; WGCNA, weighted gene co‐expression network analysis.

WGCNA identified six gene modules in GSE66272. Among them, the turquoise module exhibited the strongest positive correlation with the immunometabolism score and comprised 1457 genes (Figure 1A,B). Differential expression analysis in TCGA‐KIRC revealed 986 immunometabolism‐related DEGs (Figure 1C). Intersection analysis yielded 22 shared genes for downstream analysis (Figure 1D). ssGSEA indicated a marked enrichment of activated B cells, CD4^+^ T cells, CD8^+^ T cells, dendritic cells, NK cells, and macrophages in KIRC tissues compared to controls (Figure S2).

### 3.2 Construction and validation of the diagnostic model

**FIGURE 2:**
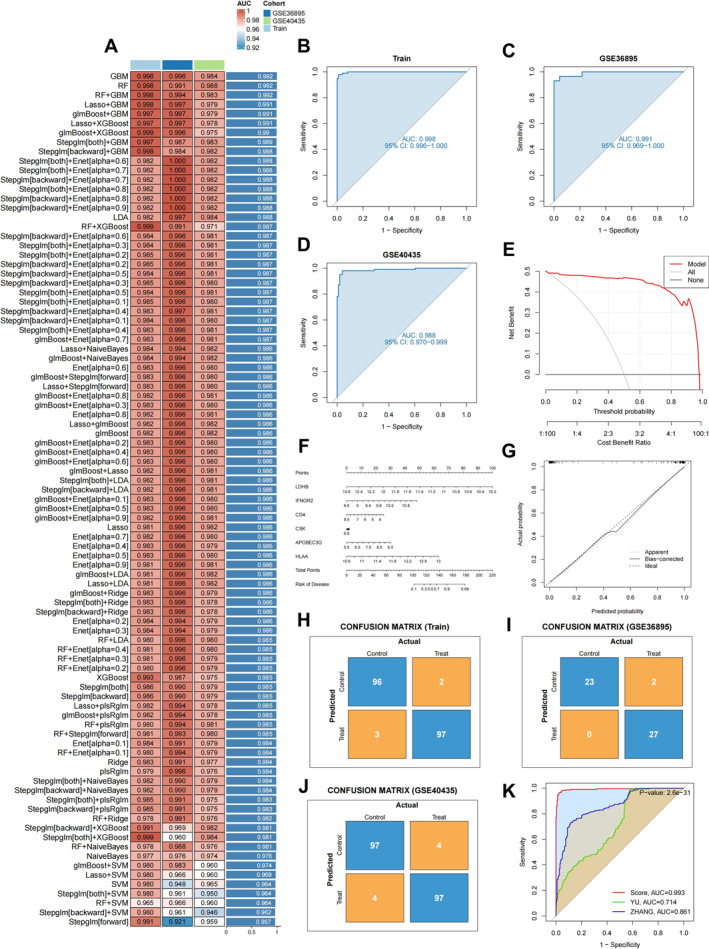
Construction and validation of the diagnostic model. (A) Schematic of the diagnostic model construction. (B–D) ROC curves for the training and validation cohorts. (E–G) Calibration curves of the diagnostic model. (H–J) Confusion matrices for the training and validation cohorts. (K) Comparison of ROC curves between the proposed diagnostic model and alternative models. ROC, receiver operating characteristic.

A total of 113 algorithmic combinations, generated from 12 machine learning methods, were evaluated via 10‐fold cross‐validation using 22 overlapping candidate genes (Figure 2A). Overfitted and large‐gene models were excluded. The final RF model selected six genes: APOBEC3G, CD4, CSK, HLA‐A, IFNGR2, and LDHB. ROC analysis showed high predictive performance with AUCs of 0.998 (training), 0.991 (GSE36895), and 0.988 (GSE40435) (Figure 2B–D). DCA indicated clinical benefit across the full threshold range (Figure 2E). The diagnostic nomogram (Figure 2F) demonstrated excellent calibration (Figure 2G), and confusion matrices confirmed true positive and negative rates >0.9 in all cohorts (Figure 2H–J). Compared to published KIRC models (AUC = 0.714) ^28^ (AUC = 0.861),^29^ the RF model showed superior performance (AUC = 0.993, *p* = 2.6e‐31) (Figure 2K).

### 3.3 Enrichment analysis of diagnostic model‐related genes

**FIGURE 3:**
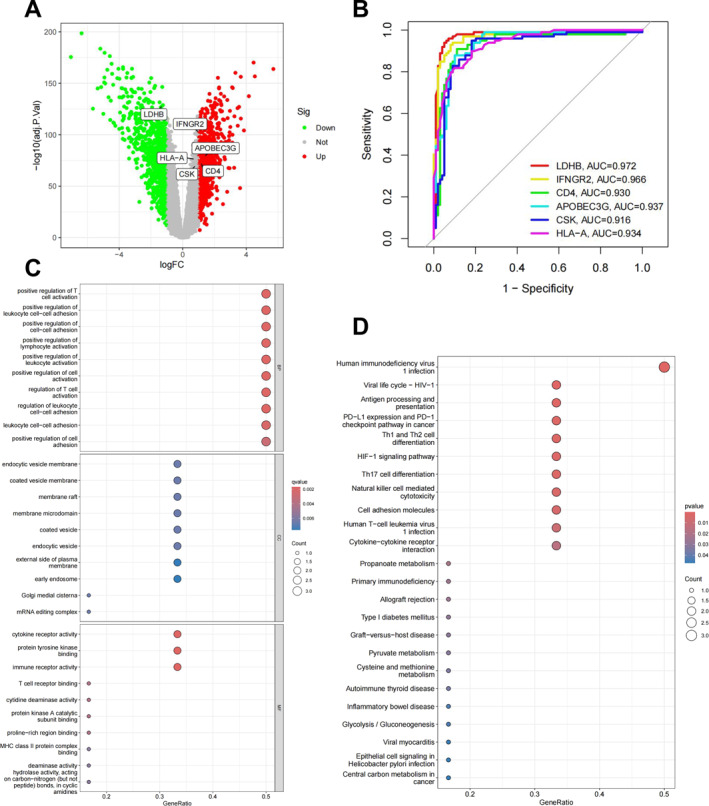
Enrichment analysis of diagnostic model‐related genes. (A) Volcano plot of diagnostic model‐related genes. (B) Receiver operating characteristic curves of diagnostic model‐related genes. (C) Gene Ontology analysis. (D) Kyoto Encyclopedia of Genes and Genomes pathway analysis.

A volcano plot visualization of genes from the RF diagnostic model was generated (Figure 3A). ROC analysis showed that all six genes (LDHB, IFNGR2, CD4, CSK, HLA‐A, and APOBEC3G) had strong diagnostic performance (AUC >0.9), with LDHB achieving the highest (AUC = 0.972) (Figure 3B). GO analysis revealed enrichment in immune cell activation (biological process), endocytic vesicle membranes (cellular component), and cytokine receptor activity (molecular function) (Figure 3C). KEGG analysis indicated involvement in antigen presentation, PD‐1/PD‐L1 checkpoint, Th1/Th2 differentiation, and HIF‐1 signaling pathways (Figure 3D).

### 3.4 Single‐cell analysis

**FIGURE 4:**
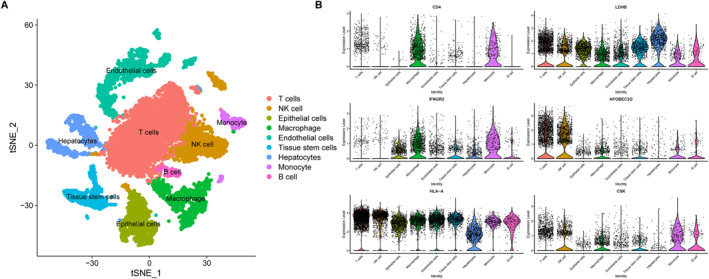
Single‐cell analysis. (A) Cell types identified in KIRC tissues based on the GSE171306 dataset. (B) Expression levels of random forest diagnostic model‐related genes in the identified cell types from KIRC tissues. KIRC, kidney renal clear cell carcinoma.

Functional enrichment analysis of genes derived from the RF diagnostic model revealed a significant association with immune processes. To investigate the association between these genes and immune cells, single‐cell analysis was performed on the GSE171306 dataset. Based on gene expression profiles, cells were classified into nine distinct populations (Figure 4A): T cells (*n* = 3924), NK cells (*n* = 1568), epithelial cells (*n* = 904), macrophages (*n* = 1002), endothelial cells (*n* = 1630), tissue stem cells (*n* = 814), hepatocytes (*n* = 932), monocytes (*n* = 641), and B cells (*n* = 209). The relationship between the RF diagnostic model‐related genes and these nine cell types is shown in Figure 4B. CD4 was significantly enriched in macrophages and monocytes; IFNGR_2_ was significantly enriched in epithelial cells, macrophages, and monocytes; APOBEC3G was primarily enriched in T cells and NK cells; CSK was highly enriched in monocytes, B cells, and NK cells; and HLA‐A and LDHB were significantly enriched across all nine cell types.

### 3.5 Drug sensitivity analysis

**FIGURE 5:**
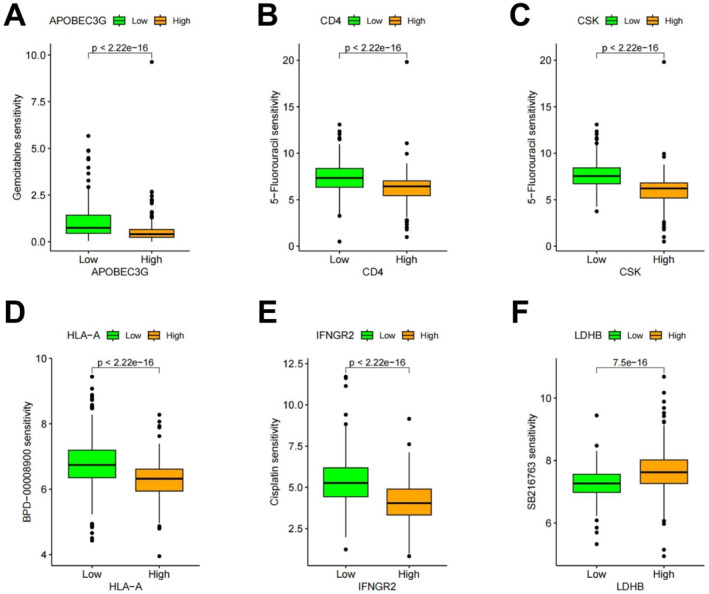
Drug sensitivity analysis of diagnostic model‐related genes. (A) Drug sensitivity analysis of APOBEC3G. (B) Drug sensitivity analysis of CD4. (C) Drug sensitivity analysis of CSK. (D) Drug sensitivity analysis of HLA‐A. (E) Drug sensitivity analysis of IFNGR_2_. (F) Drug sensitivity analysis of LDHB. CSK, Cytoskeleton‐Associated Protein; LDHB, Lactate Dehydrogenase B.

**FIGURE 6:**
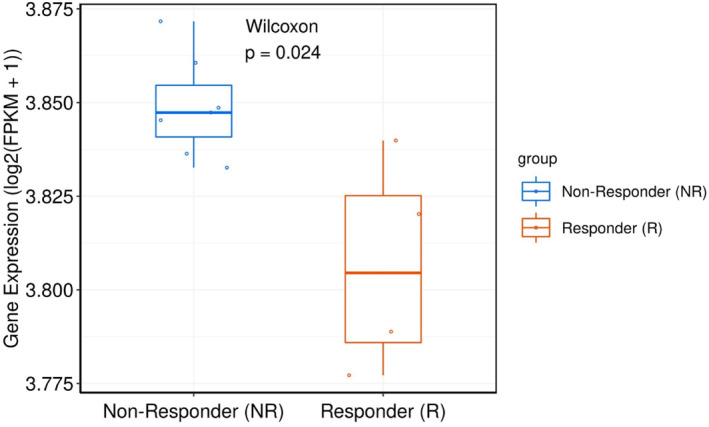
Sensitivity analysis of HLA‐A to PD‐L1. Boxplot showing HLA‐A expression levels (log_2_(FPKM + 1)) in PD‐L1 therapy responders (R) and non‐responders among kidney renal clear cell carcinoma patients. Statistical significance was assessed using the Wilcoxon rank‐sum test (*p* = 0.024).

Drug response profiling of RF model‐associated genes demonstrated that gemcitabine downregulated APOBEC3G in KIRC, whereas 5‐fluorouracil inhibited CD4 and CSK expression. Pembrolizumab suppressed HLA‐A, cisplatin reduced IFNGR2, and SB216763 upregulated LDHB, collectively contributing to KIRC inhibition (Figure 5). Moreover, PD‐L1 sensitivity analysis revealed significantly lower HLA‐A expression in patients who responded favorably to PD‐1 therapy compared to non‐responders (Figure 6).

### 3.6 Differential expression analysis of diagnostic model‐related genes

**FIGURE 7:**
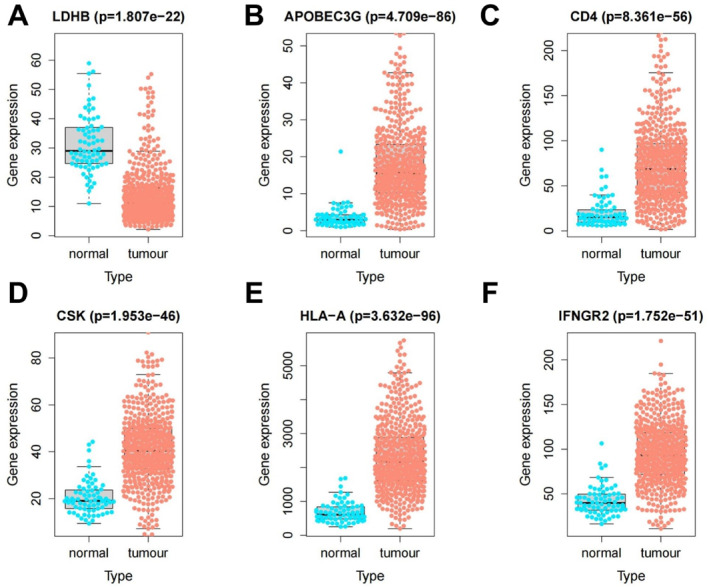
Expression patterns of diagnostic model‐related genes. (A) LDHB expression is significantly lower in KIRC tissues compared to normal tissues. (B–F) APOBEC3G, CD4, CSK, HLA‐A, and IFNGR_2_ expression is significantly higher in KIRC tissues than in normal tissues. Statistical analysis was performed using the Wilcoxon rank‐sum test, with *p* < 0.05 indicating significant differences. CSK, Cytoskeleton‐Associated Protein; KIRC, kidney renal clear cell carcinoma; LDHB, Lactate Dehydrogenase B.

To investigate expression patterns of genes linked to the KIRC diagnostic model, we first examined six key genes in KIRC and normal tissues using the TCGA database (Figure 7). The analysis showed that LDHB expression was markedly reduced in KIRC tissues relative to normal tissues (Figure 7A), whereas IFNGR_2_, CD4, CSK, HLA‐A, and APOBEC3G were significantly upregulated (Figure 7B–F).

**FIGURE 8:**
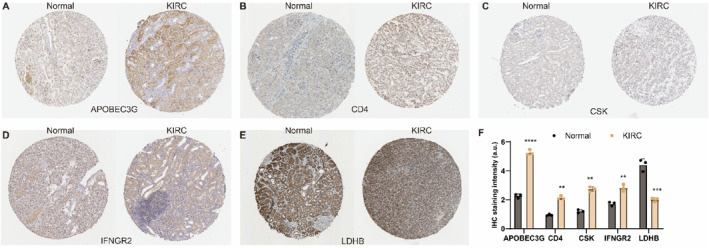
Immunohistochemical validation of diagnostic model‐related genes. (A) Immunohistochemical staining of APOBEC3G (antibody: HPA001812). (B) Immunohistochemical staining of CD4 (antibody: HPA004472). (C) Immunohistochemical staining of CSK (antibody: HPA026488). (D) Immunohistochemical staining of IFNGR_2_ (antibody: HPA001535). (E) Immunohistochemical staining of LDHB (antibody: CAB004641). The left side shows normal kidney tissues (*n* = 3) and the right shows KIRC tissues (*n* = 3). Staining was performed using DAB (3,3′‐diaminobenzidine) to visualize protein expression levels (brown signals). All images were captured at ×4 magnification. (F) Quantification of IHC staining intensity of APOBEC3G, CD4, CSK, IFNGR2, and LDHB in normal kidney and KIRC tissues (*n* = 3 per group). Data are presented as mean ± standard deviation. Statistical analysis was conducted using the *t*‐test. **p < 0.01, ***p < 0.001, ****p < 0.0001. CSK, Cytoskeleton‐Associated Protein; IHC, Immunohistochemistry; KIRC, kidney renal clear cell carcinoma; LDHB, Lactate Dehydrogenase B.

To further validate these expression trends, we analyzed the protein expression of the six genes in normal renal tubular epithelial cells and KIRC cells using the HPA database (Figure 8A–F). Immunohistochemical data showed reduced LDHB levels in KIRC compared to normal renal tubular epithelial cells, whereas IFNGR_2_, CD4, CSK, and APOBEC3G expression was higher in KIRC tissues, aligning with the mRNA trends in TCGA. It is worth noting that immunohistochemical data for HLA‐A were unavailable, necessitating further confirmation of its protein expression through additional experimental approaches.

### 3.7 Validation of expression patterns of six key genes

**FIGURE 9:**
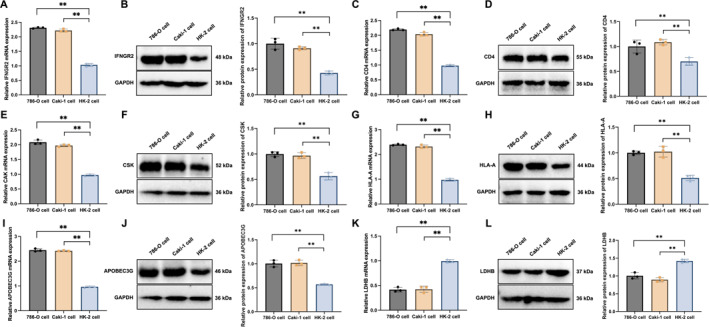
mRNA and protein expression levels of six key genes in kidney renal clear cell carcinoma cell lines. (A, C, E, G, I, and K) Quantitative real‐time polymerase chain reaction analysis of APOBEC3G, CD4, CSK, HLA‐A, IFNGR2, and LDHB expression, respectively. (B, D, F, H, J, and L) Corresponding western blot analysis of the same six genes. Comparisons were conducted among 786‐O and Caki‐1 renal carcinoma cells and normal human renal tubular epithelial cells (HK‐2). All experiments were performed in triplicate (*n* = 3). Data are presented as mean ± standard deviation. Statistical analysis was conducted using the *t*‐test. *p < 0.05, **p < 0.01. CSK, Cytoskeleton‐Associated Protein; LDHB, Lactate Dehydrogenase B.

To validate the expression trends of the six key diagnostic genes (LDHB, IFNGR2, CD4, CSK, HLA‐A, and APOBEC3G) identified in the diagnostic model, qRT‐PCR and western blot analyses were performed in KIRC cell lines (786‐O and Caki‐1) and normal renal tubular epithelial cells (HK‐2) (Figure 9).

qRT‐PCR results (Figure 9A, C, E, G, I, K) showed that IFNGR2, CD4, CSK, HLA‐A, and APOBEC3G were significantly upregulated in both KIRC cell lines (all *p* < 0.01), whereas LDHB was markedly downregulated. Western blot results (Figure 9B, D, F, H, J, L) corroborated these findings, demonstrating increased protein levels of the five upregulated genes and a marked decrease in LDHB protein levels in KIRC cells relative to HK‐2 cells (all *p* < 0.01).

### 3.8 Expression and functional validation of six key genes in KIRC cells

To evaluate the regulatory efficiency of the key genes and their impact on KIRC cell behavior, IFNGR2, CD4, CSK, HLA‐A, and APOBEC3G were silenced, and LDHB was overexpressed in 786‐O and Caki‐1 cells. Both qRT‐PCR and western blot analyses confirmed that silencing the five oncogenic genes significantly reduced their mRNA and protein expression levels by more than 70% (*p* < 0.01), whereas LDHB expression was significantly increased by approximately 80% following overexpression (*p* < 0.01) (Figure S3A,B).

Functional assays revealed that knockdown of the five genes or overexpression of LDHB markedly inhibited the proliferation, migration, and invasion of KIRC cells and induced apoptosis (*p* < 0.01, Figure S3C–F). Consistent trends were observed in both cell lines.

### 3.9 In vivo validation and functional roles of six key genes in a xenograft model

In xenograft models using 786‐O cells, knockdown of IFNGR2, CD4, CSK, HLA‐A, and APOBEC3G, and overexpression of LDHB were confirmed by qRT‐PCR and western blot, with modulation efficiencies exceeding 70% and 80%, respectively (*p* < 0.01) (Figure S4A–D). All treatment groups displayed a significant reduction in tumor size and weight relative to controls, with inhibition rates over 50% (*p* < 0.01) (Figure S4E,F). H&E staining showed decreased cellular density, whereas Immunohistochemistry (IHC) revealed lower Ki‐67 and higher cleaved‐caspase 3 levels (Figure S4G–J). The knockdown groups exhibited stronger tumor suppression than the LDHB overexpression group (*p* < 0.05).

## 4 DISCUSSION

KIRC represents the most prevalent subtype of renal malignancies in adults. Owing to its asymptomatic onset, it is frequently diagnosed at an advanced stage or after metastasis, resulting in an unfavorable prognosis.^30^ Despite growing research, reliable biomarkers for early detection and personalized treatment remain scarce, limiting diagnostic sensitivity and contributing to substantial heterogeneity in therapeutic response.^31^ By integrating transcriptomic data from the TCGA and GEO databases and applying WGCNA and 12 machine learning algorithms, six immunometabolism‐related diagnostic biomarkers (LDHB, IFNGR2, CD4, CSK, HLA‐A, and APOBEC3G) were identified. A nomogram‐based diagnostic model constructed from these markers achieved AUCs exceeding 0.98 across training and external validation cohorts, significantly outperforming previously published models and demonstrating promising clinical applicability.

Although some of these genes have been reported in other cancer types, their expression patterns and functional roles in KIRC exhibit notable differences. LDHB, a key enzyme in lactate metabolism, has been implicated in enhancing glycolytic activity and driving tumor cell proliferation across various cancer types.^32^, ^33^, ^34^ However, LDHB is markedly downregulated in KIRC, suggesting a potential tumor‐suppressive role in this context. IFNGR2, a critical subunit of the interferon‐gamma receptor, is known to enhance immune responses and improve immunotherapy efficacy in melanoma and other tumors.^35^, ^36^ In KIRC, IFNGR2 is positively correlated with immune cell infiltration and better prognosis. CD4, a classical marker for helper T cells, has been linked to immunotherapy responsiveness.^14^ Its upregulation in KIRC and association with immune cell infiltration further support its relevance in immune surveillance.

CSK negatively regulates T Cell Receptor (TCR) signaling via suppression of SRC Proto‐Oncogene, Non‐Receptor Tyrosine Kinase (SRC) family kinases.^37^, ^38^ Its downregulation in KIRC may contribute to dysregulated immune responses and the development of a hyperactive TME. Although its roles in migration and signaling have been studied in other cancers, data in KIRC remain scarce.^39^, ^40^ HLA‐A, a core component of the MHC‐I complex, is essential for antigen presentation and has been linked to mechanisms of tumor immune escape.^14^ In KIRC, its expression is negatively correlated with PD‐L1, suggesting an independent function in maintaining immune recognition. APOBEC3G, a DNA/RNA editing enzyme commonly associated with high mutational burden and tumor evolution,^41^, ^42^ shows a strong positive correlation with the infiltration of B cells, CD8^+^ T cells, and dendritic cells in KIRC, implying a possible role in enhancing adaptive immune responses.

These six markers collectively participate in metabolic regulation, immune activation, and antigen presentation in KIRC, indicating complementary and potentially synergistic functions. APOBEC3G is positively associated with PD‐L1 levels and may promote both immunogenicity and immune escape.^43^ LDHB downregulation is associated with poor prognosis and reduced immune infiltration,^44^ possibly through modulation of lactate metabolism and the TME. It also influences lysosomal function and autophagy,^45^ whereas LDHA‐generated lactate has been shown to suppress IFN‐γ secretion from T cells and NK cells, facilitating immune evasion.^46^ CD4, IFNGR2, and CSK are predominantly expressed in T‐cell subsets and are involved in immune activation and regulation of TCR signaling. HLA‐A is expressed in both dendritic cells and tumor cells, regulated by IFN‐γ, and is essential for antigen presentation.^14^

Single‐cell transcriptomic profiling validated the specific enrichment of these genes in T cells, B cells, CD8^+^ T cells, NK cells, and dendritic cells,^47^ supporting their central roles in the immune microenvironment of KIRC. CD4, IFNGR2, and CSK regulate T‐cell function; HLA‐A and IFNGR2 coordinate antigen presentation and interferon signaling; APOBEC3G mediates cytotoxic responses; and LDHB links metabolic status to immune suppression. Collectively, these findings delineate a convergent “metabolism–immunity–antigen presentation” axis that offers mechanistic insight into immune evasion, metabolic reprogramming, and persistent inflammation in KIRC, potentially informing the development of dual‐targeted therapeutic strategies.

Despite the well‐defined roles of these markers in KIRC, their functions in other renal cancer subtypes, such as Kidney Renal Papillary Cell Carcinoma (KIRP) and Kidney Chromophobe (KICH), remain largely unexplored. APOBEC3G has been linked to immune suppression and unfavorable prognosis in KIRC,^43^ suggesting possible immunoregulatory potential in other subtypes. IFI16, a protein within the interferon pathway, is differentially expressed and linked to immune infiltration in KIRP and KICH,^48^ underscoring the broader relevance of interferon signaling, although IFNGR2 has not been specifically studied. Limited evidence exists for the roles of LDHB, IFNGR2, CD4, CSK, and HLA‐A in KIRP or KICH. Future studies involving multi‐omics analyses and cross‐subtype comparisons are warranted to clarify their expression profiles, immune associations, and clinical significance in other renal carcinomas, thus evaluating their potential as pan‐RCC biomarkers.

Clinically, the diagnostic model enables rapid, interpretable risk assessment by integrating key gene expression profiles. Patients with low LDHB and high IFNGR2 or APOBEC3G expression may exhibit stronger immune activity and greater immunotherapy sensitivity. Furthermore, multiple identified markers demonstrate responsiveness to targeted therapies such as sorafenib and pembrolizumab, highlighting their value in predicting therapeutic response. Experimental validation confirms their biological relevance, supporting future combination strategies integrating metabolic and immune‐based therapies.

Despite the relatively comprehensive and in‐depth findings of this study, certain limitations must be considered. First, the data sources were primarily derived from public databases (TCGA and GEO). Although external validation cohorts were used, prospective validation using real‐world clinical samples from multiple centers and diverse populations is still lacking. Second, although machine learning approaches effectively uncover hidden patterns in high‐dimensional data, differences in algorithm selection, parameter tuning, and training strategies may influence the final biomarker selection and introduce a potential risk of model overfitting. Moreover, although the experimental validations encompassed qRT‐PCR, western blot, and in vivo animal models, mechanistic investigations for some genes remain limited. Future studies should incorporate more in‐depth approaches such as CRISPR‐based gene knockout, chromatin immunoprecipitation, and protein–protein interaction profiling to further elucidate their regulatory networks and functional roles.

To facilitate real‐world clinical application, future efforts should focus on validating the robustness and generalizability of the diagnostic model across large‐scale, multicenter prospective cohorts. Simultaneously, integrating spatial transcriptomics with immunohistochemical analyses may offer valuable insights into the spatial architecture and cell–cell communication networks of key immune‐related genes in KIRC tissues. A broader multi‐omics approach, encompassing metabolomics, proteomics, and epigenomics, could further refine the characterization of the immunometabolic landscape. Additionally, leveraging artificial intelligence and deep learning algorithms may optimize diagnostic accuracy and enable intelligent automated clinical decision support. Ultimately, these advances aim to bridge the gap between research and practice by fostering the development of practical diagnostic solutions, including molecular assay kits, liquid biopsy platforms, and personalized therapeutic decision‐making tools.

## 5 CONCLUSION

This study identified six novel immunometabolism‐related diagnostic biomarkers for KIRC, LDHB, IFNGR2, CD4, CSK, HLA‐A, and APOBEC3G, using integrative transcriptomic analysis and machine learning. These biomarkers effectively distinguish tumors from normal tissues, exhibit strong associations with immune cell infiltration and clinical characteristics, and are significantly enriched in key immune cell populations. A diagnostic nomogram constructed using these markers outperformed existing models in predictive performance. Drug sensitivity, single‐cell, and in vivo experiments further confirmed their biological and therapeutic relevance. Together, our findings offer clinically translatable biomarkers and a foundation for early diagnosis and personalized therapy in KIRC.

## AUTHOR CONTRIBUTIONS

G.H. conceived the study, performed data analysis, and drafted the manuscript. J.L., M.F., and H.L. contributed to data acquisition, bioinformatics analysis, and figure preparation. J.H. supervised the project, provided critical revisions, and secured funding. All authors interpreted the data, approved the final manuscript, and agreed to be accountable for the work.

## CONFLICT OF INTEREST STATEMENT

The authors declare no conflicts of interest.

## ETHICS STATEMENT

All animal experiments were approved by the Animal Ethics Committee of Yangjiang People's Hospital. We ensure that all efforts were made to minimize animal suffering and to adhere to the highest standards of animal welfare throughout the research process.

## Supporting information

Supporting Information S1

## Data Availability

All data generated or analyzed during this study are included in this article and/or its supplementary material files. Further inquiries can be directed to the corresponding author.
