# Correction to an immunometabolism‐related signature for renal clear cell carcinoma diagnosis and therapeutic target

**DOI:** 10.1002/ccs3.70097

**Published:** 2026-07-16

Hu, Guofan, Jian Liang, Meiling Feng, Hansheng Lin, and Jingwei He. 2025. “An Immunometabolism‐Related Signature for Renal Clear Cell Carcinoma Diagnosis and Therapeutic Target.” *Journal of Cell Communication and Signaling*: e70047. https://doi.org/10.1002/ccs3.70047.

In the original article, the incorrect version of the abstract was published. The correct version is published below. The online version of the article has been updated.

We apologize for this error.

Abstract

Kidney renal clear cell carcinoma (KIRC) lacks sensitive biomarkers for early diagnosis and effective guidance for personalized therapy. Given the close interplay between tumor metabolism and immune regulation, this study aimed to identify immunometabolism‐related biomarkers for KIRC and develop a robust diagnostic model. Transcriptomic datasets from TCGA and GEO were integrated, and immunometabolism‐related genes were collected from GeneCards. Weighted gene co‐expression network analysis (WGCNA) and differential expression analysis identified candidate genes, which were subsequently evaluated using 12 machine learning algorithms with 113 algorithmic combinations. The optimal model was selected through 10‐fold cross‐validation and validated in independent external cohorts. Additional analyses included immune infiltration, functional enrichment, single‐cell RNA sequencing, drug sensitivity, and PD‐L1 response assessments, while gene expression and biological functions were verified through TCGA, HPA, qRT‐PCR, Western blotting, and experimental studies.

A total of 22 overlapping immunometabolism‐related genes were identified. Among the 113 algorithmic combinations, the random forest model achieved the best performance and identified six key biomarkers: APOBEC3G, CD4, CSK, HLA‐A, IFNGR2, and LDHB. The diagnostic model demonstrated excellent predictive accuracy, with AUC values of 0.998 in the training cohort, 0.991 in GSE36895, and 0.988 in GSE40435. These biomarkers were significantly associated with immune cell infiltration and enriched in immune‐related pathways, including antigen presentation, PD‐1/PD‐L1 signaling, Th1/Th2 differentiation, and HIF‐1 signaling. Single‐cell analysis revealed distinct enrichment patterns across immune cell populations, while drug sensitivity analysis suggested potential interactions with gemcitabine, 5‐fluorouracil, pembrolizumab, cisplatin, and SB216763. Expression validation showed that IFNGR2, CD4, CSK, HLA‐A, and APOBEC3G were upregulated, whereas LDHB was downregulated in KIRC. Functional experiments demonstrated that silencing IFNGR2, CD4, CSK, HLA‐A, or APOBEC3G, or overexpressing LDHB, significantly inhibited proliferation, migration, and invasion and promoted apoptosis in KIRC cells. In xenograft models, these interventions also markedly suppressed tumor growth.

Overall, six immunometabolism‐related biomarkers were identified and integrated into a high‐performing diagnostic model for KIRC. These biomarkers are closely associated with the immune microenvironment and tumor biology and may serve as promising candidates for early diagnosis and personalized therapeutic targeting in KIRC.

