# Supplementary material for: An immunometabolism‐related signature for renal clear cell carcinoma diagnosis and therapeutic target
Source: J Cell Commun Signal. 2025 Sep 17;19(3):e70047. doi: 10.1002/ccs3.70047 (PMC12443813; doi:10.1002/ccs3.70047)
Supplement: Supplementary file 1 — Supporting Information S1 [file CCS3-19-e70047-s001.docx]

**Table S1. Dataset/Queue Information and Usage.**

| **Dataset/Queue** | **Detailed Information** | **Usage** |
| --- | --- | --- |
| GSE66272 | 27 KIRC tissue samples and 27 normal kidney tissue samples | WHCNA analysis, training set |
| GSE53757 | 72 KIRC tissue samples and 72 normal kidney tissue samples | Training set |
| GSE36895 | 30 KIRC tissue samples and 24 normal kidney tissue samples | Test set |
| GSE40435 | 101 KIRC tissue samples and 101 normal kidney tissue samples | Test set |
| GSE171306 | 2 KIRC tissue samples | Single-cell analysis |
| TCGA-KIRC | 542 KIRC tissue samples and 72 normal kidney tissue samples | Extraction of immune-metabolic related genes |

**Table S2. Primer Sequences for qPCR Analysis.**

| **Gene** | **Primer Sequence (5'-3')** |
| --- | --- |
| IFNGR2 | Forward: 5'-CTCCATTCTGCCTGGGTGACAA-3'  Reverse: 5'-CGTGGAGGTATCAGCGATGTCA-3' |
| CD4 | Forward: 5'-CCTCCTGCTTTTCATTGGGCTAG-3'  Reverse: 5'-TGAGGACACTGGCAGGTCTTCT-3' |
| CSK | Forward: 5'-AAGGTGGAGCACTACCGCATCA-3'  Reverse: 5'-AGTCCATCTGCGTCTGAGGTGT-3' |
| HLA-A | Forward: 5'-AGATACACCTGCCATGTGCAGC-3'  Reverse: 5'-GATCACAGCTCCAAGGAGAACC-3' |
| APOBEC3G | Forward: 5'-ATGACACCTGGGTCCTGCTGAA-3'  Reverse: 5'-GAATCACGTCCAGGAAGCACAG-3' |
| LDHB | Forward: 5'-GGACAAGTTGGTATGGCGTGTG-3'  Reverse: 5'-AAGCTCCCATGCTGCAGATCCA-3' |
| GAPDH | Forward: 5'-GTCTCCTCTGACTTCAACAGCG-3'  Reverse: 5'-ACCACCCTGTTGCTGTAGCCAA-3' |

**Table S3. Primary and Secondary Antibodies Used for Western Blot Analysis.**

| **Antibody** | **Molecular Mass** | **Catalog Number** | **Manufacturer** |
| --- | --- | --- | --- |
| anti- IFNGR2 antibody | 38 kDa | ab171081 | Abcam |
| anti- CD4 antibody | 51 kDa | ab133616 | Abcam |
| anti- CSK antibody | 50 kDa | ab125005 | Abcam |
| anti- HLA-A antibody | 41 kDa | ab52922 | Abcam |
| anti- APOBEC3G antibody | 46 kDa | ab302926 | Abcam |
| anti- LDHB antibody | 37 kDa | ab53292 | Abcam |
| GAPDH antibody | 36 kDa | 60004-1-Ig | Proteintech |

**Table S4. shRNA Sequences Targeting CXCL10 and MC4R.**

| **Gene** | **Primer sequence (5'-3')** |
| --- | --- |
| shRNA- IFNGR2 | Forword: 5'- TGCTGCTGATGACATCGTTT-3' |
|  | Reverse: 5'- AAACGATGTCATCAGCAGCA-3' |
| shRNA-CD4 | Forword: 5'- GGCGTATCTGTGTGAGGACT-3' |
|  | Reverse: 5'- AGTCCTCACACAGATACGCC-3' |
| shRNA-CSK | Forword: 5'- CATTAAACCAAAGGTCATGG-3' |
|  | Reverse: 5'- CCATGACCTTTGGTTTAATG-3' |
| shRNA-HLA-A | Forword: 5'-GGAGATCACACTGACCTGGC-3' |
|  | Reverse: 5'-GCCAGGTCAGTGTGATCTCC-3' |
| shRNA-APOBEC3G | Forword: 5'-GCTGCTGATGACATCGTTTA-3' |
|  | Reverse: 5'-TAAACGATGTCATCAGCAGC-3' |
| shRNA-control | Forword: 5'-TTCTCCGAACGTGTCACGT-3' |
|  | Reverse: 5'- ACGTGACACGTTCGGAGAA-3' |

**
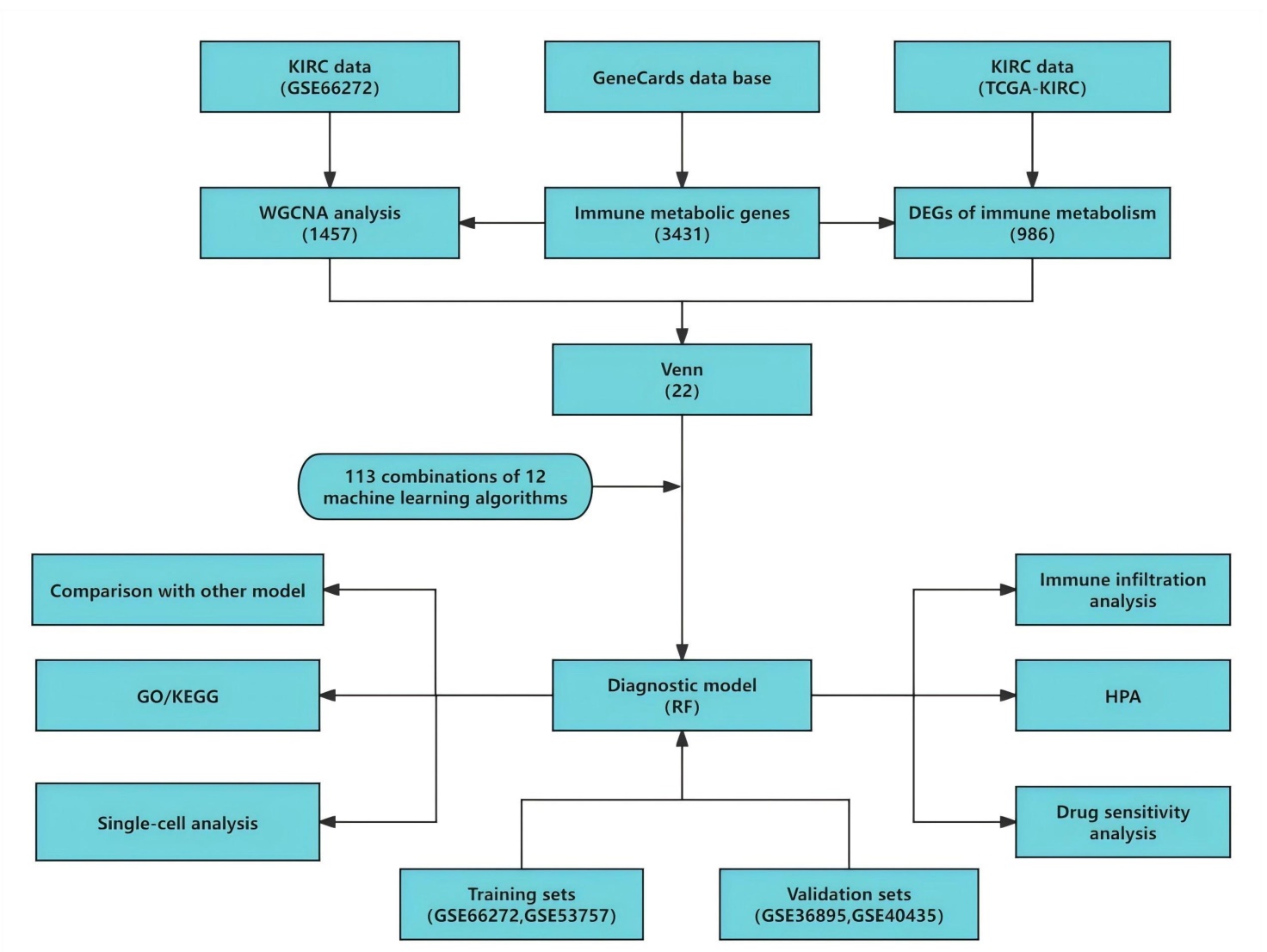
**

**Figure S1. Flowchart of the Study Design.**

**
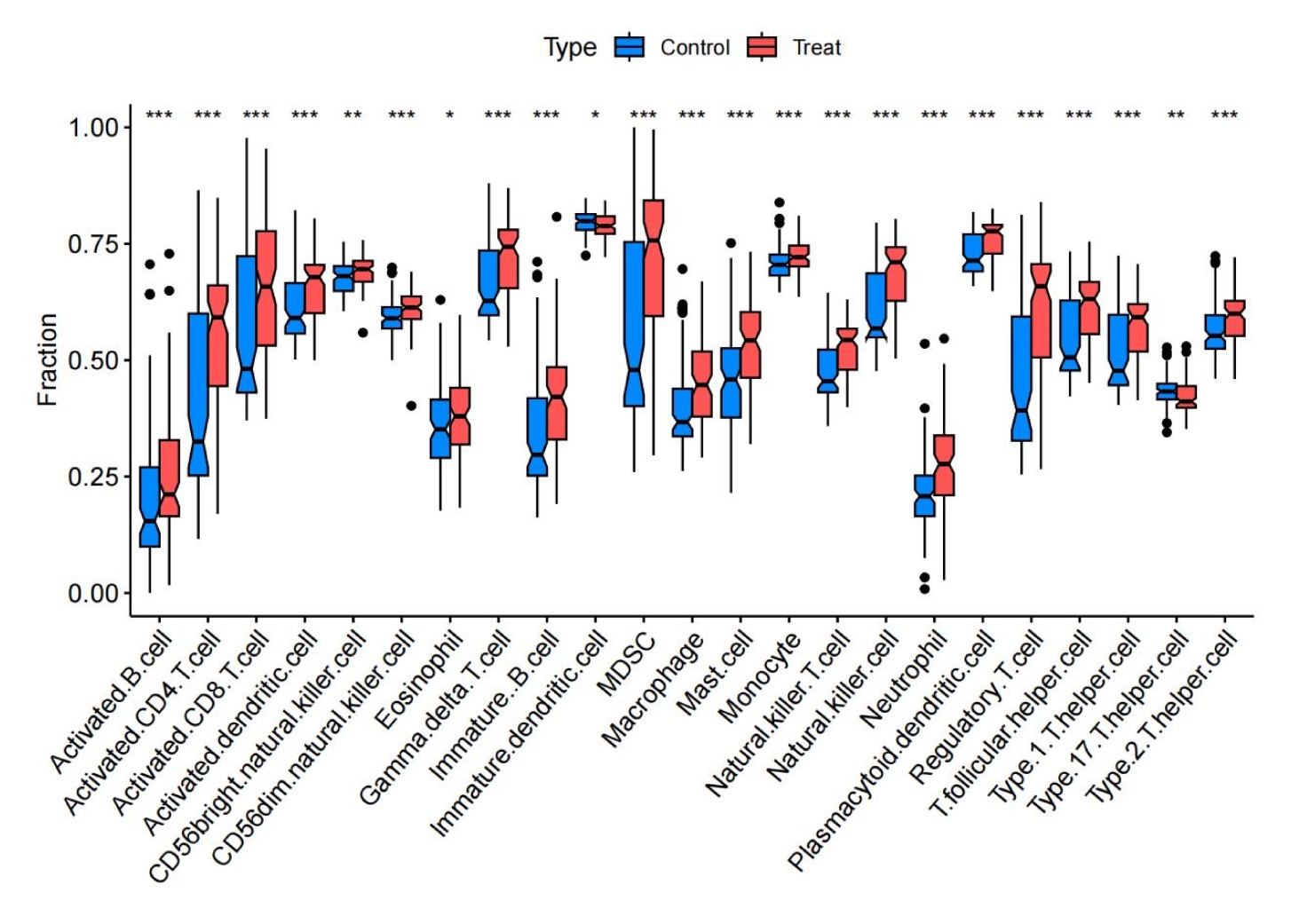
**

**Figure S2. Comparison of Immune Cell Subset Abundances Between Control and Treatment Groups in KIRC.**

**
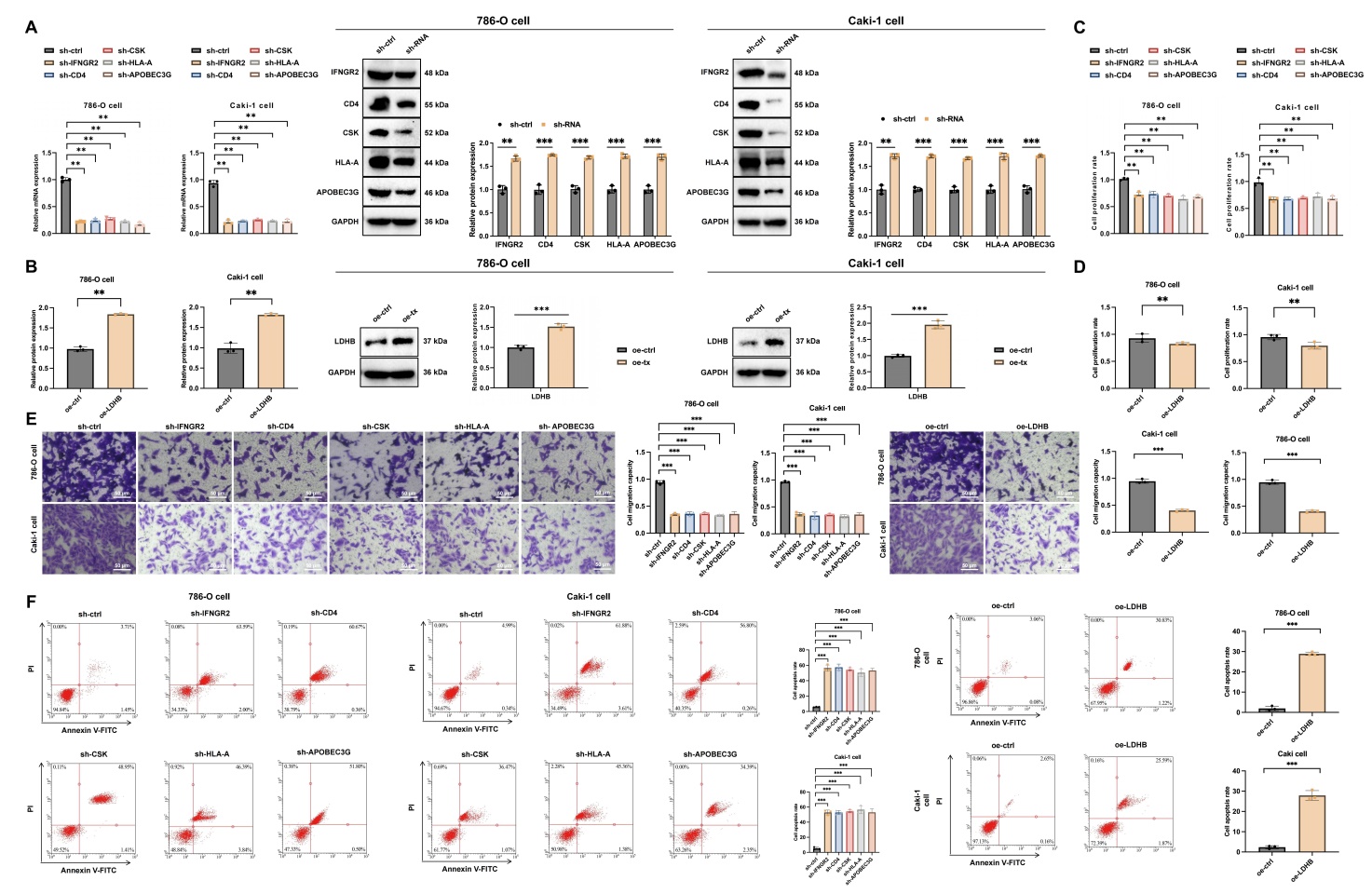
**

**Figure S3. Effects of Silencing or Overexpressing Six Key Genes on the Biological Functions of KIRC Cells.**

Note: (A–B) qRT-PCR and Western blot were used to validate the knockdown efficiency of IFNGR2, CD4, CSK, HLA-A, and APOBEC3G, as well as the overexpression efficiency of LDHB; (C–D) CCK-8 assay was performed to assess the effects of gene knockdown or overexpression on KIRC cell proliferation; (E) Transwell assay was used to evaluate the effects of different gene interventions on the migration and invasion abilities of KIRC cells, including representative microscopic images and quantitative bar graphs; (F) Annexin V/PI flow cytometry was used to detect apoptosis rates following gene manipulation. All experiments were performed in triplicate. Data are presented as mean ± standard deviation. Each target gene was knocked down individually; no combined knockdown was performed. Statistical significance was determined using the t-test; **p* < 0.05, ***p*< 0.01.

**
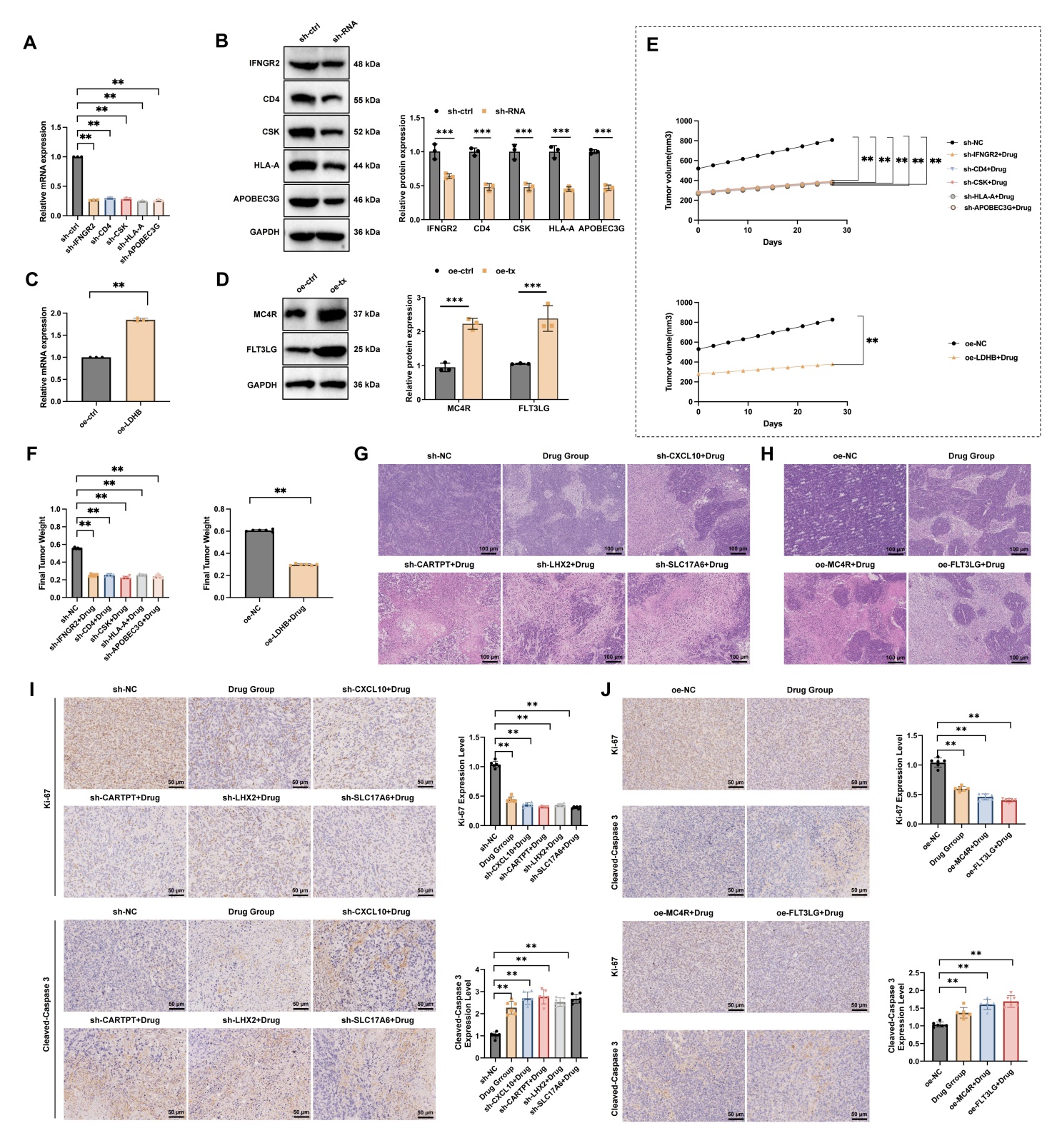
**

**Figure S4. *In vivo* Validation and Functional Effects of Six Key Genes in a Xenograft Model.**

Note: (A–D) qRT-PCR and Western blot were used to validate the knockdown efficiency of IFNGR2, CD4, CSK, HLA-A, and APOBEC3G, as well as the overexpression efficiency of LDHB; (E–F) Tumor volume growth curves and final tumor weight analysis in different gene intervention groups in the xenograft model; (G–H) H&E staining was performed to evaluate structural changes in tumor tissues from the knockdown and overexpression groups; (I–J) IHC analysis of Ki-67 and Cleaved-Caspase 3 expression levels in the knockdown and overexpression groups, with representative microscopic images and quantitative bar graphs shown. All experiments were conducted in triplicate. Each target gene was knocked down individually; no combined knockdown was performed. Data are presented as mean ± standard deviation; **p* < 0.05, ***p* < 0.01; N = 6.
